# Raman imaging reveals in-situ microchemistry of cuticle and epidermis of spruce needles

**DOI:** 10.1186/s13007-021-00717-6

**Published:** 2021-02-08

**Authors:** Nadia Sasani, Peter Bock, Martin Felhofer, Notburga Gierlinger

**Affiliations:** Department of Nanobiotechnology (DNBT), Institute for Biophysics, University of Natural Resources and Life Sciences (BOKU), Muthgasse 11-II, 1190 Vienna, Austria

**Keywords:** Cuticle, Waxes, Epidermis, Norway spruce, Confocal Raman microscopy, Non-negative matrix factorization, Cluster analysis, Microchemistry

## Abstract

**Background:**

The cuticle is a protective layer playing an important role in plant defense against biotic and abiotic stresses. So far cuticle structure and chemistry was mainly studied by electron microscopy and chemical extraction. Thus, analysing composition involved sample destruction and the link between chemistry and microstructure remained unclear. In the last decade, Raman imaging showed high potential to link plant anatomical structure with microchemistry and to give insights into orientation of molecules. In this study, we use Raman imaging and polarization experiments to study the native cuticle and epidermal layer of needles of Norway spruce, one of the economically most important trees in Europe. The acquired hyperspectral dataset is the basis to image the chemical heterogeneity using univariate (band integration) as well as multivariate data analysis (cluster analysis and non-negative matrix factorization).

**Results:**

Confocal Raman microscopy probes the cuticle together with the underlying epidermis in the native state and tracks aromatics, lipids, carbohydrates and minerals with a spatial resolution of 300 nm. All three data analysis approaches distinguish a waxy, crystalline layer on top, in which aliphatic chains and coumaric acid are aligned perpendicular to the surface. Also in the lipidic amorphous cuticle beneath, strong signals of coumaric acid and flavonoids are detected. Even the unmixing algorithm results in mixed endmember spectra and confirms that lipids co-locate with aromatics. The underlying epidermal cell walls are devoid of lipids but show strong aromatic Raman bands. Especially the upper periclinal thicker cell wall is impregnated with aromatics. At the interface between epidermis and cuticle Calcium oxalate crystals are detected in a layer-like fashion. Non-negative matrix factorization gives the purest component spectra, thus the best match with reference spectra and by this promotes band assignments and interpretation of the visualized chemical heterogeneity.

**Conclusions:**

Results sharpen our view about the cuticle as the outermost layer of plants and highlight the aromatic impregnation throughout. In the future, developmental studies tracking lipid and aromatic pathways might give new insights into cuticle formation and comparative studies might deepen our understanding why some trees and their needle and leaf surfaces are more resistant to biotic and abiotic stresses than others.

## Background

Norway spruce (*Picea abies*) is the most abundant tree species in forests of the European Alps. High biomass accumulation, straight growth and a satisfactory rejuvenation account for its high popularity in forestry. Trees are exposed to tough environmental conditions and various abiotic and biotic stresses, which assigns a key role to the needle cuticle as first line of defense [1–6]. It is composed of waxes and lipids [7]. The main function is to prevent the loss of water to the atmosphere, enable mechanical protection and to mitigate abiotic and biotic stresses such as UV light, changing relative humidity, temperature and microorganisms [4, 8–12]. A wealth of studies regarding cuticle chemistry, mechanics and functions is available. However, most of these studies used more or less destructive methods to study the cuticle. For description, SEM [13–21] and TEM [14, 16, 22–28] were extensively used, while chemistry was mostly examined after extraction procedures [21, 29–48]. These extraction and washing procedures destroy the native structure of the cuticle and do not allow insights into the spatial distribution of cuticle components. Recently, several reviews recognized this shortcoming and expressed the need for in-situ methods to link chemical with spatial information [49–51]. Yet, only a few studies use methods like confocal laser scanning microscopy [52, 53], IR or Raman microscopy [13, 54–56]. Unfortunately, none of these studies showed detailed information about the cuticular layers and their respective chemistry.

In this study, we show high resolution Raman images depicting the composition of the cuticle in the needles of Norway spruce (*Picea abies*). Microsections were mapped at two excitation wavelengths and polarization measurements were conducted to probe the alignment of the molecules with respect to the plant surface. The mappings include the cuticle together with the epidermal layer beneath and reveal chemical heterogeneity using univariate as well as multivariate data analysis.

## Results

**Fig. 1:**
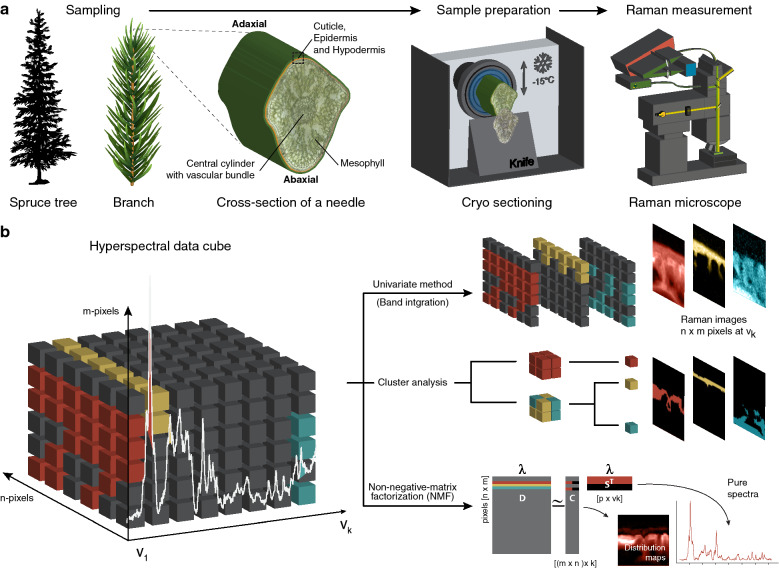
Raman imaging of native plant cuticles. **a** After sampling the needles are kept frozen, also during cutting of microsections with the cryo-microtome. Cross sections (20 µm thick) are scanned using a Confocal Raman microscope. The region of interest included the adaxial cuticle together with the underlying epidermal layer. **b** The resulting hyperspectral data cube is analysed using three different approaches: Univariate band integration, clustering spectra based on similarity and retrieving the most pure components based on Non-negative-matrix-factorizations

**Fig. 2:**
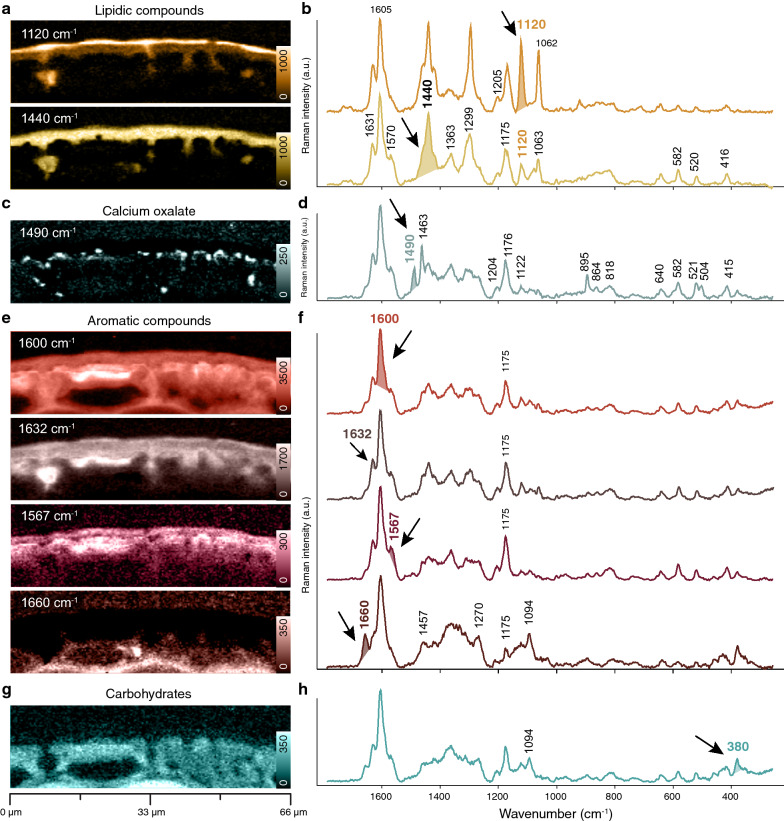
Raman imaging of the cuticle of Norway spruce based on band integration and extracted average spectra of the visualized chemically different regions **a** The outer lipidic layers are visualized by integrating the band at 1120 cm^−1^ and 1440 cm^−1^ and **b** the extracted average spectra confirm the lipidic character, but also include bands attributed to aromatics. **c** Integrating the band at 1490 cm^−1^ visualizes a pointwise accumulation of Calcium oxalate **d** as confirmed by the bands at 1490, 1463 and 895 cm^−1^. **e** Integrating different aromatic bands shows their distribution throughout the cuticle and epidermal layer and **f** extracted average spectra confirm that different aromatic components play a role **g** Integrating the characteristic cellulose band at 380 cm^−1^ displays the lower cuticle and **h** the derived average spectrum confirms carbohydrates together with aromatics

**Fig. 3:**
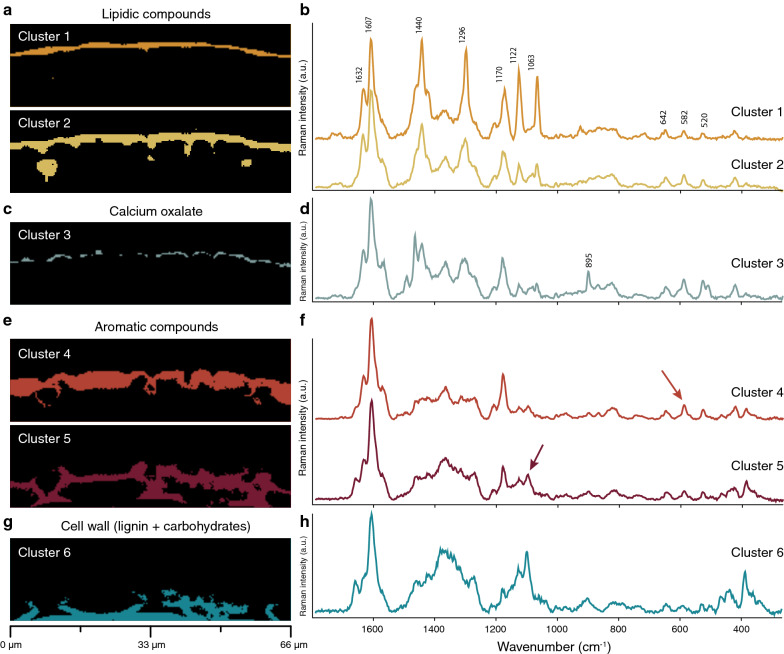
Cluster analysis performed on the hyperspectral dataset of spruce cuticle (same as shown in Fig. 2). **a**, **b** Two outer lipidic clusters (1 and 2) are distinguinshed. **c**, **d** Subclustering the lower lipidic layer displays the Calcium oxalate accumulation (see also Additional file 1: Fig. S1). **e–h** Epidermal layer is divided into three clusters based on changes in amount and composition of aromatic components and carbohydrates

**Fig. 4:**
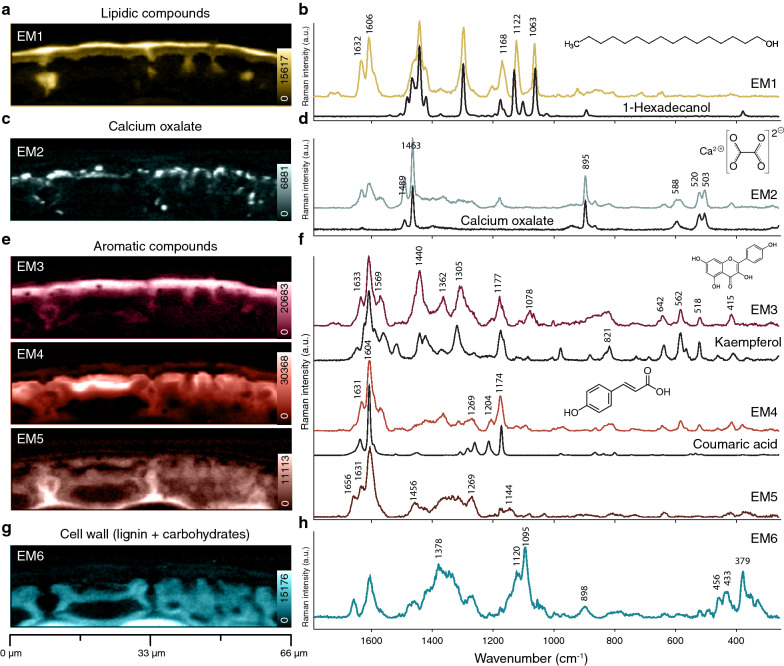
Non-negative matrix factorization analysis of the cuticle of Norway spruce. **a–h** Shown are abundance maps depicting the distribution of the six end members. End members spectra are compared with spectra of pure reference compounds. The brightest color corresponds to the highest intensity

**Fig. 5:**
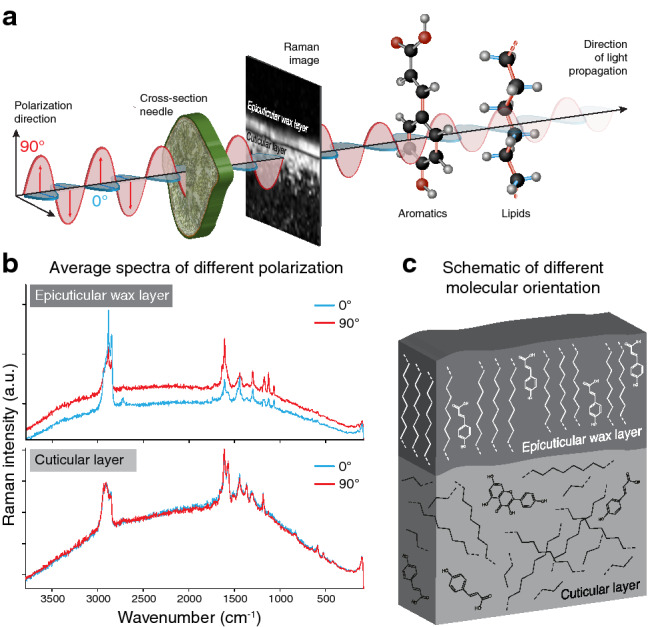
Probing molecule orientation in spruce cuticle by mapping with different laser polarization. **a** The same region of interest was mapped with laser polarization direction parallel (0°) and perpendicular (90°) with respect to the cuticle surface **b** Raman spectra are extracted selectively from the epicuticular wax layer and the underneath cuticular layer. In the top layer higher intensities are observed for C**–**H stretches of waxes (2900 cm^−1^) at 0° laser polarization (blue spectrum), while C**–**C stretching of waxes and aromatic ring stretches are most intense with 90° (red spectrum). In the cuticular layer beneath no orientation dependence is observed as the spectra are identical. **c** Schematic representation of the orientation of waxes and coumaric acid in the cuticle. The aliphatic chains and aromatic rings are oriented perpendicular to the surface in the wax layer, while compounds in the cuticular layer are not oriented, comprising an amorphous structure

This study sheds new light on spruce cuticles by using high-resolution (~ 300 nm) confocal Raman spectroscopy (CRM). Cutting 20 µm thick microsections of the needles with a cryo-microtome enabled to scan pointwise across the native cuticle and including the underlying epidermal layer. Based on the acquired Raman spectra (hyperspectral data cube), chemical images were generated using univariate as well as multivariate data analysis (Fig. 1). Plotting the peak intensity of selected Raman bands (Fig. 2), grouping the Raman spectra based on their similarity using cluster analysis (Fig. 3) and retrieving the purest chemical components using the unmixing approach Non-negative matrix factorization (NMF) (Fig. 4) revealed the chemically different regions and the corresponding Raman spectra. The endmember spectra from non-negative matrix factorization (NMF) were compared with spectra of pure references to proof the assignment of the Raman bands. Due to high sample fluorescence (with 532 nm), we used 785 nm excitation for the Raman imaging experiments. But we also include 532 nm measurements, which show the potential of laser polarization to retrieve preferred alignments of the molecules in the outer region of the cuticle (Fig. 5).

### Integrating specific Raman bands to highlight chemical differences

The very outer epicuticular wax layer is displayed by integrating the Raman band at 1120 cm^−1^, while the whole cuticle shows up by integrating the CH_2_ bend at 1440 cm^−1^ [57] (Fig. 2a). The spectrum of the outermost epicuticular wax layer shows two pronounced bands at 1122 cm^−1^ and 1062 cm^−1^ (Fig. 2b). The sharpness of these bands is indicative of a crystalline, highly ordered state. Below the cuticle two sharp bands are discovered at 1490 cm^−1^ and 1463 cm^−1^ and their integration displays pointwise accumulations of Calcium oxalate deposits (Fig. 2c, d), as Calcium oxalate monohydrate (Caox) shows strong Raman bands at 1498 cm^−1^, 1474 cm^−1^ and 902 cm^−1^ [58]. In all spectra of the cuticle (Fig, 2b, d) a strong band is found at 1603 cm^−1^, which can be attributed to aromatic ring stretching vibrations [59]. Integration of this band reveals that aromatic components play a role in the cuticle as well as in the underlying epidermal layer (Fig. 2e, f). Integrating the neighboring band at 1632 cm^−1^ highlights the upper cuticle and protrusions towards the epidermal layer (Fig. 2e). Integrating the other aromatic bands at 1567 cm^−1^ and 1660 cm^−1^, displays the upper and lower epidermal layer, respectively (Fig. 2e). Hence, the different integrations and derived average spectra (Fig. 2e, f) show that aromatics are found in all layers, but their composition changes. The spectra of the epidermal layers (Fig. 2f) show additionally bands at e.g. 380 cm^−1^ and 1094 cm^−1^, which can be assigned to cellulose [60]. Integrating the 380 cm^−1^ band depicts the epidermal layer and plant cell wall spectra are derived with strong aromatic bands at 1600 cm^−1^ and 1175 cm^−1^ (Fig. 2g, h). Integration of the 860 cm^−1^ band is commonly used for visualization of pectin [61], but in these spectra this band is too weak or overlapped by other components to be used for pectin imaging.

### Multivariate approaches: analyzing all bands at once

#### Cluster analysis

Multivariate data analysis methods have the advantage to analyze the whole wavenumber region (hyperspectral data cube) at once, instead of focusing on selected bands. Cluster analysis extracts pixels based on their spectral similarity and displays chemically similar regions (clusters) and their average spectra (Fig. 3). Based on the results by band integration, a division into more than four clusters (water, waxes, cuticle, epidermal layer) to detect differences in chemistry within the cuticle and epidermal layer was expected. From the analysis based on four to eight clusters, we finally show the results based on seven clusters (Fig. 3 and Additional file 1: Fig. S1) to include the most chemically different regions. The waxy layer on the upper side (cluster 1) is clearly distinguished from the lower cuticle (cluster 2) (Fig. 3a, b). Within the cuticle the Calcium oxalate deposits are included and only differentiated by further subclustering of the lower cuticular layer (Additional file 1: Fig. S1). The average spectrum shows beside the Calcium oxalate bands also bands attributed to aromatics and lipids and the distribution seems more layer like (Fig. 3c, d). Within the epidermal layer three clusters are separated with decreasing intensity of the aromatic 1175 cm^−1^ contribution and increasing signal of the carbohydrates (1095 cm^−1^, 380 cm^−1^) (Fig. 3c, d). Cluster 5 represents the lower epidermal layer and shows a typical secondary cell wall spectrum with carbohydrates and aromatics. All retrieved cluster average spectra include many different bands and components: in the upper layers, lipids are mixed with aromatics, below these, Calcium oxalate is mixed with lipids and aromatics and in the epidermal layer on the bottom, aromatics are mixed with carbohydrates (Fig. 3).

### Non-negative-matrix factorization (NMF)

In a next step, an unmixing algorithm, Non-negative-matrix factorization (NMF) was applied to retrieve the purest spectral signatures of the different components together with their distribution [62]. These purest component spectra are called endmember (EM) spectra and are compared to spectra of reference compounds to verify components and Raman band assignments (Fig. 4 and Table 1). The outer epicuticular wax layer of the cuticle is distinguished by EM1 and the spectrum includes typical bands of crystalline waxes (doublet at 1122 and 1063 cm^−1^) as also observed in solid 1-hexadecanol (Fig. 4a, b). EM2 displays calcium oxalate as almost all bands match with a spectrum of pure calcium oxalate (Fig. 4c, d). Accumulations are often pointwise and mainly below the lipidic layer and a few protrusions in between the epidermal cells-similar to that observed by band integration (Fig. 2c, d). EM3 represents the lipidic layer below the wax layer and the spectrum shows beside lipid bands strong aromatic signals, which partly coincide with Kaempferol (Fig. 4e, f). EM4 highlights the upper epidermal layer and the strongest aromatic bands coincide with the bands of coumaric acid. EM 4 and EM 5 were mutually excluding each other. EM 5 reflects lignin and shows highest concentration in the middle lamella between the epidermal cells. EM6 finally displays secondary cell walls of the epidermal cells with strong carbohydrate bands (1378 cm^−1^, 1120 cm^−1^, 1095 cm^−1^) and less lignin (1600 cm^−1^) (Fig. 4g, h). Overall, the unmixing method results in more pure component spectra, but still aromatic contributions are revealed in all of them, reflecting the intimate mixing of aromatics with lipids and carbohydrates.

**Table 1:** Assignment of different components of the cuticle. Wavenumbers derived from NMF

Wavenumber (cm^−1^)			Assignment
Wax	Cutin	CaOx	
1734	1723		ν C=O (fatty acid esters)
1712	1707		ν C=O (coumaric acid, midchain carbonyls)
	1654		ν C=C (anthoxanthins)
1632	1634		ν C=C (coumaric acid, stilbenes); Φ8 (anthoxanthins)
1607	1607		Φ8 (all rings)
	1570		ν C=O (anthoxanthins)
		1488	ν C=O (Calcium oxalate) [107]
		1463	ν C=O (Calcium oxalate) [107]
1455			δ C–H (aliphatic chains) [57]
1441	1440		δ C–H (aliphatic chains) [57]
1423			δ C–H (aliphatic chains) [57]
1372			γ_w_ CH_2_ (aliphatic chains) [57]
	1362		Φ20a (anthoxanthins(A-ring))
	1308		γ_t_ CH_2_ (aliphatic chains) [57]
1295			γ_t_ CH_2_ [57]
1201	1205		Φ7a (coumaricacid)
	1177		Φ9a (anthoxanthins with p-subst. C-Ring)
1169			Φ9a (coumaric acid)
1123			ν C–C (aliphatic chains)
	1112		
	1077		ν C–C (aliphatic chains)
1062	1066		ν C–C (aliphatic chains)
	1001		Φ12 (stilbenes)
921			ν C–C (aliphatic chains)
		895	ν_s_ C–C + δ O–C=O (Calcium oxalate) [107]
864	870	864	ν C–C + γ_r_ CH_2_ (aliphatic chains) [108]; (Calcium oxalate)
	831		ν C–C + γ_r_ CH_2_ (aliphatic chains) [108]
	738		(Kaempferol)
710			
653			
642	642		Φ6a (anthoxanthins (A-ring))
		593	Water libration (Calcium oxalate) [107]
	582		Φ1 (anthoxanthins (A-ring))
524		520	ν Ca-O + ν C–C (Calcium oxalate) [107]
	518		Φ6b (anthoxanthins (A-ring))
		503	
418	414		

ν: stretching; δ: bending; γ_t_: twisting; γ_r_: rocking, γ_w_: wagging; Φ: ring mode in Varsanyi notation [59]

### Polarization dependent intensity changes probe molecular orientation

Most Raman microscopes work with linear-polarized lasers. Acquiring spectra with different laser polarization direction (0° and 90° with respect to the sample/molecule orientation, Fig. 5a) detects whether chemical components are ordered or not. Two subsequent measurements were run on the same area: one image scan with the laser polarization aligned in parallel to the cuticle of the needle and the other perpendicular to it (Fig. 5a). As the 532 nm laser can induce artefacts in the spectra of subsequent measurement [62], mappings were started with either parallel or perpendicular laser polarization to estimate any potential damage. Although a notable increase in background attributed to the second measurement was seen in the spectra (Fig. 5b), there were no signs of laser degradation. Regardless of which polarization was used first, the spectra differed in the same way between polarization runs. The spectra of the outermost wax layer differ with respect to parallel (0°) and perpendicular (90°) laser polarization. The C–H stretches of waxes (~ 2900 cm^−1^) were captured when the laser polarization was parallel (red spectrum), while C–C stretching of waxes and aromatic ring stretches were most intense with perpendicular orientation (blue spectrum) (Fig. 5b). A similar orientation dependence was recorded on neat fatty acids (see Additional file 1: Fig. S2), showing either the C–H stretching (~ 2900 cm^−1^) or "in-line" modes (CH_2_-twisting, C–C stretching) intensified if chains are aligned parallel to each other and the laser. In the cuticular layer underneath no polarization dependence was observed. The spectra were identical (Fig. 5b), proofing also that no laser damage occurred. Thus, orientation of lipids and aromatics is proven in the epicuticular wax layer, while in the layer underneath no preferred alignment of the molecules is detected (Fig. 5c).

## Discussion

**Fig. 6:**
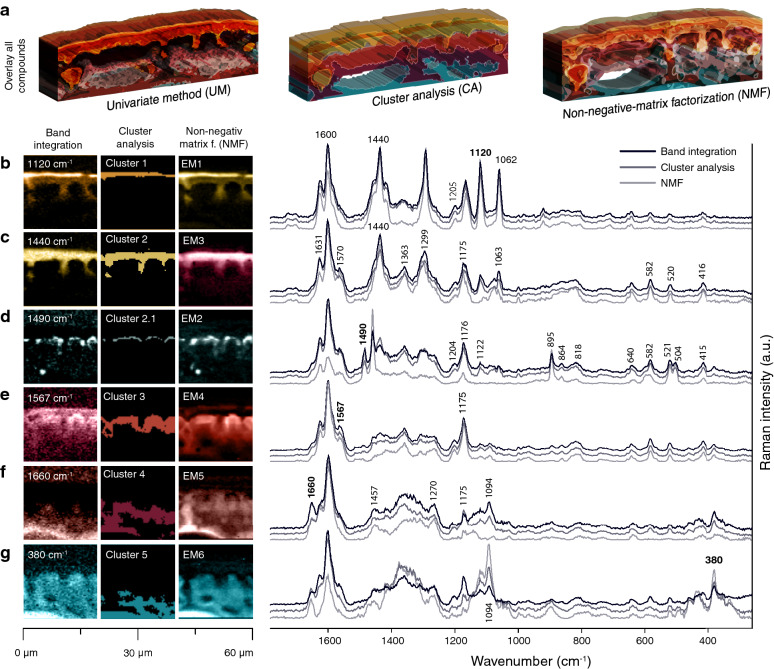
Comparison of the three different methods (Band integration, Cluster analysis, and Non-negative-matrix factorization (NMF)). **a** Overlay of all compounds from each method. The Raman images were rasterized, and 3D extruded for better visualizing the overlaying nature of the different compounds. **b–g** Sections of the Raman images (Figs. 2, 3, 4) from each method to compare each extracted layer. On the right the corresponding spectra

Assessing cuticle chemistry by techniques like NMR, gas chromatography or mass spectrometry requires depolymerization prior to analysis and the native cuticle structure is destroyed [63]. For a complete understanding of this outer protective layer more in-situ methods and studies are needed to reveal the chemistry in context with microstructure [49–51]. In this work, we show that confocal Raman microscopy probes the cuticle in the native state and gives access to aromatics, lipids, carbohydrates and minerals at once. The acquired hyperspectral dataset is the basis to image the chemical heterogeneity using univariate- and multivariate data analysis (Fig. 6). Mapping with changed laser polarization direction even probes the orientation of the molecules with respect to the plant surface (Fig. 5). One major advantage of Raman point-by-point mapping is the fact that one has not to rely only on “images”. Behind every pixel is a molecular fingerprint, and average, cluster and endmember spectra help to interpret and verify the chemical composition of distinguished layers, interfaces and agglomerations.

### Which aromatic components are represented in cuticle Raman spectra?

The phenolic nucleus gives rise to strong Raman bands, which can be used to image the distribution of aromatics along the whole cuticle and epidermis. The strongest band at 1605 cm^−1^ is present throughout the whole cuticle and epidermis. It indicates aromatic rings in conjugation with C=C/C=O [64, 65]. In needles, coumaric acid, stilbenes and flavonoids are reported [66–69]. To assign specific bands to these different aromatics and track them selectively, a critical survey of reference spectra is necessary (Additional file 1: Fig. S3–S9). Coumaric acid has prominent bands at 1636, 1606 and 1171 cm^−1^, which have also been detected in EM4 (Fig. 4e, f and Table 1). Comparing the EM4 spectrum with different coumaric acid derivatives (Additional file 1: Fig. S3), we can clearly assign it to coumaric acid. Our interpretation therefore is that the majority of coumaric acid is present as individual molecules and that only minor parts may be esterified. The band at 1175 cm^−1^ represents aromatic CH bending of para-substituted rings (Φ9a) and is therefore not unique to coumaric acid. Benzoic acid and its derivates show this band as well as flavonoids with para-substituted C-rings. Of all substitution variants, only 4-methoxybenzoic acid achieves a satisfactorily overlap (see Additional file 1: Fig. S4).

The strong Raman band around 1570 cm^−1^ serves as a marker band for anthoxanthins (flavonoids) [70] and can be found in the whole cuticle (Fig. 2e, f, EM3 in Fig. 4e, f), except for the epicuticular wax layer. While the band at 1175 cm^−1^ hints to a para-substituted C-Ring (Φ_para_ 9a), the bands at 642 (Φ_asym-tetra_ 6a), 582 (Φ_asym-tetra_ 1), and 520 cm^−1^ (Φ_asym-tetra_ 6b) show an A-ring with two hydroxyl groups (Table 1). Such a flavone would be kaempferol and its reference spectrum matches well with the EM3 spectrum (Fig. 4e, f, and Additional file 1: Fig. S5). The spectrum of ( +)-Catechin, the flavanol we tested, cannot be matched with the cuticle spectra. Stilbenes, previously found in needles of spruce (piceatannol, astringin or isorhapontin) [66, 71] have a characteristic Raman line at 1000 cm^−1^ (Φ_sym-tri_ 12) [57, 72]. Interestingly, spectra of pinosylvin and pinosylvin monomethylether can be matched best, although these are not reported for spruce needles, while those of resveratrol, piceatannol, isorhapontigenin and astringin show additional bands and based on these cannot be fitted to the cuticle spectra (see Additional file 1: Fig. S6). Stilbenes have large Raman cross-sections enabling their identification even in small amounts [72]. Due to only a weak band at 1000 cm^−1^ being present in the cuticle spectra, we conclude that the amount of stilbenes must be rather low. Also picein and piceol were found in spruce needles in comparatively high amounts [66], but can be fitted to the spectra only with low intensity. Dehydroabietic acid cannot be fitted to the spectrum at all, and therefore does not occur in the needle’s cuticle (see Additional file 1: Fig. S7).

Lignin is well separated from cuticle-specific phenolics by the band at 1660 cm^−1^ (Figs. 2e, f, 4e, f) which is attributed to lignin monolignols [64, 73]. Contrary to [74], we do not regard the band at 1630 cm^−1^ as a lignin band, instead we primarily assign it to coumaric acid (ethylenic C=C stretch) (see Table 1).

### Epicuticular waxes align with coumaric acid perpendicular to the surface

Epicuticular waxes build the outer layer of plant cuticles to prevent transpiration and water loss [7]. They are composed of long-chain aliphatic compounds with several functional groups (e.g. hydroxyls and esters). Hydroxy derivatives of nonacosan-10-ol, e.g. nonacosane-4, 10-diol, nonacosane-7, 10-diol or nonacosane-10, 13-diol have been identified in needle waxes from various conifers, e.g. *Picea abies* [75], *Pinus radiata* [37] or *Juniperus scopularum* [47]. Epicuticular waxes can appear film-like or as crystalloids [76], but always in their solid crystal forms [16, 17]. The Raman spectra of the epicuticular wax layer (Fig. 2a, b: 1120 cm^−1^, Fig. 3a, b: cluster 1, and Fig. 4a, b: EM1) show sharp bands typical for crystalline substances with limited degrees of rotational freedom [57]. The characteristic doublet at 1122 and 1063 cm^−1^ is only visible in solid wax (see Additional file 1: Fig. S8). Reference spectra of C_16_ and C_18_ alcohols match well, yet this does not confirm the chain length because aliphatic chains with similar carbon counts display similar spectra. The epicuticular wax bands are always observed together with aromatic bands, even in the EM spectrum based on the unmixing NMF-algorithm (Fig. 4, EM1). The strong bands at 1632 and 1606 cm^−1^ are of aromatic origin and the bands at 1201 and 1169 cm^−1^ point to coumaric acid. Such hydroxycinnamic acids protect the underlying tissue by absorbing UV-light [77]. This and the fact that we revealed even a preferred alignment of the aromatic rings along with the aliphatic chains (Fig. 5) suggests strong association of aromatics and waxes.

To derive the orientation of molecules with respect to the laser polarization direction is an unparalleled advantage of Raman microscopy. The polarizability of a normal mode is anisotropic and therefore differs with the incident angle of the electromagnetic field [57, 59] (Fig. 5a). Laser polarization experiments have been used to estimate the orientation of cellulose fibrils in the cell wall [78, 79] as well as to reveal different orientations of a lignin monomer [64]. In this study, polarization measurements show a clear orientation of waxes in the epicuticular layer, but no orientation preference in the subjacent cuticle layer (Fig. 5b, c). A model of perpendicular oriented waxes with respect to the surface of the cuticle is shown in [76]. Based on Raman we show that this orientation can actually be found in the native cuticle and moreover that aromatic rings are oriented the same way (Fig. 5c). The bands at 1600 and 1173 cm^−1^ are more intense when the laser is oriented perpendicular to the surface (Fig. 5b). In para-substituted rings, both modes Φ8a and Φ9a have the greatest polarizability change along the line connecting both substituent atoms, so that this result clearly demonstrates that the ring, and therefore coumaric acid, is aligned parallel to the aliphatic chains in the wax layer (Fig. 5c).

### Amorphous cuticle layer is impregnated with aromatics

Cutin is a polymer created from saturated hydroxylated aliphatic acids, usually a mixture of C_16_ and C_18_ ω-hydroxyl fatty acids [80, 81]. Midchain hydroxyl or epoxy groups are reported as well as additional endgroups like aldehyde, ketone and carboxyl [82–85]. In addition, glycerol, glyceryl esters, coumaric and ferulic acids have been reported [86–89]. This results in a wide variety of chemical types depending on organ (leaf or fruit), location (adaxial or abaxial surfaces of the same leaf) and stages of maturity [51, 85, 89]. The Raman spectrum of the cutin layer shows the expected bands for fatty acids (1440 and 1305 cm^−1^), which appear broader than the corresponding peaks of waxes in the overlying layer. Sharp bands indicating crystallinity are missing (Fig. 2a, b: 1440 cm^−1^, Fig. 3a, b: cluster 2, Fig. 4e, f: EM3, and Additional file 1: Fig. S8) and polarization measurements yield similar spectra (Fig. 5b). This suggests aliphatic chains in a multitude of conformations without any preferential orientation. Indeed, cutins can be viewed as a non-ordered mesh with cavities filled by other cuticle components [63]. Such components can be phenolics as confirmed by aromatic bands found in the cutin layer (Fig. 4e, f, EM3). The marker band for flavonoids at 1570 cm^−1^ is most pronounced in this layer (Fig. 6b, 2nd row) and Kaempferol was found to match the spectrum very well (Fig. 4f and Additional file 1: Fig. S5). A weak signal of stilbenes is also observed, but their actual structure remains unclear. Lipid spectra include always aromatic bands, corroborating the idea that clusters of aromatics are inserted into the cutin network [90, 91].

### Calcium oxalate accumulates at the interface between cuticle and epidermal layer

Calcium oxalate crystals exist in many plants and they appear in many tissues and organs. They are diverse in shape, size, number of crystals and hydration [92]. They play various roles such as cation regulation, CO_2_ and H_2_O supply, tissue support, herbivore protection, detoxification, and light manipulation [93–103].

In spruce needles, calcium oxalate was found in vascular bundles, in intercellular spaces, inside of cell walls and as many tiny pure calcium oxalate crystals in the cuticular layer [96, 104]. Our Raman approach detects these crystals in a layer-like fashion below the cutin layer (Fig. 6b, third row). The EM3 spectrum proves with sharp bands at 1490, 1463, 895 and 503 cm^−1^ calcium oxalate monohydrate as the main component (Figs. 4c, d, 6b), but still aromatic bands are present from the surrounding tissue. The crystals enhance light transmission and probably most of the “pure” crystals are smaller than 600 nm, which is about the limit of depth resolution. The Calcium oxalate crystals are mainly at the interface between the lipidic cuticle and the aromatic rich upper epidermal layer, but some are also visualized in the lumen of the epidermal cells (Fig. 6b).

### Outer epidermal cell wall: enhancing protection by aromatics

Bound flavonoids and their derivatives and other aromatics have been detected in the cell walls of the outer epidermal cell layer of spruce needles by confocal laser scanning microscopy [105]. In this work, Raman imaging reveals in the periclinal upper epidermal cell wall a strong accumulation of aromatics, which leads to a separation from the lower epidermal layer by cluster analysis and NMF (Fig. 6b). The high intensity of the aromatic band 1600 cm^−1^ (Fig. 2e) comes from the fact that coumaric acid as well as flavonoids accumulate in this region (Fig. 4, EM 4). Raman bands of cellulose together with almost zero signal of lipid components confirm the epidermis classification of this layer. In a recently published review, the authors suggest “the plant cuticle as a lipidized epidermal cell wall region” [51]. Based on our Raman imaging results the epidermal layer seems not to get “lipidized”, but “aromatized”. Thus, if this special “epidermal” layer with high accumulation of aromatics should belong to the cuticle, a definition based on coumaric acid would be necessary. Regardless of definition, our results show the importance of aromatics in linking the lipidic cuticle and carbohydrate rich epidermal layer as the same aromatic components are found in both layers. The high accumulation of flavonoids in this outwards epidermal cell wall, will enhance protection of the plant surface. Flavonoids and other aromatics in the epidermal layer of cuticles are reported to mediate a highly complex UV-screening mechanisms of Norway spruce needles [105].

### Polysaccharides detected in the epidermal layer, but hardly in the cuticle

Raman spectroscopy is sensitive to molecular vibrations of any chemical compound. However, differentiating carbohydrates in secondary plant cell walls by Raman spectroscopy can be challenging due to relatively small Raman cross sections when compared to conjugated aromatic molecules [64]. Whether carbohydrates should be regarded as authentic cuticle constituents is still debated in the field [51]. The main polysaccharides in the cuticle of Norway spruce, found by immune-gold labeling, were cellulose, mannans and pectin [27]. They were also found in similar quantities in the cuticles of tomato [14], eucalyptus, poplar and prunus [23]. Additionally, in the case of tomato, no preferred orientation or crystallinity of the polysaccharides could be found [14]. For visualization of pectin and cellulose, the Raman marker bands at 856 cm^−1^ [106] and 380 cm^−1^ can be used [61]. Pectin could not be unambiguously identified, because its marker band overlapped with another band (864 cm^−1^) probably originating from an aromatic compound. The Signal of cellulose was mainly found in the walls of the epidermal cells, but was hardly seen in the cuticle.

### Potential of Raman imaging: univariate and/or multivariate analysis?

Probing the cuticle and epidermis together, all chemical components at once and in context with the microstructure results in “comprehensive pictures” of the plant surface (Fig. 6a). The hyperspectral dataset offers many possibilities for data analysis and we show and discuss one univariate and two multivariate approaches. All three separated the wax layer from the underlying cuticle and highlighted the adjacent periclinal epidermal layer as chemically different from the rest of the epidermis (Fig. 6a).

The first approach is univariate data analysis by integrating the individual Raman bands to produce intensity-dependent heat maps and extracting average spectra based on intensity thresholds for detailed analysis (Fig. 2). As it is fast and captures well chemical heterogeneity band integration was also used in the first Raman imaging experiments on wood [15, 109] and is nowadays included in almost every Raman imaging study. On our examples band integration worked well to highlight the waxy layer on top of the cuticle based on the sharp crystalline band at 1120 cm^−1^ in a similar way to the unmixing algorithm NMF (Fig. 6b, EM1). Cluster analysis separates the wax layer (cluster 1) from the cuticle, as with this approach no intensity threshold (overlay of layers) is possible, and spectra are sorted by spectral similarity either in one or the other cluster (Fig. 6b, c). The derived cluster average spectrum reflects directly the chemistry of the displayed region, similar like average spectra derived based on band integration. On contrast, NMF-analysis calculates a set of endmember spectra, which are combined to reproduce the experimental spectra of the plant sample at every pixel [62]. The spectra of the waxy layer derived from the three approaches are very similar and include bands of lipids, but also aromatics (e.g. 1606 and 1632 cm^−1^). As the unmixing approach is not capable of finding a “pure” wax spectrum within all the pixels, we can conclude that lipids and coumaric acid are tightly intermixed. A conclusion, which would not be possible based on the other two approaches. This tight association is also seen in EM3, in which flavonoids and coumaric acids are together with lipids in the amorphous cuticle layer (Fig. 6c). These results are in full agreement with reported clusters of aromatics that are inserted into the cutin network [90, 91].

On the Calcium oxalate layer beneath, the performance of three approaches differed most (Fig. 6d). Although seven clusters have been chosen (Additional file 1: Fig. S1), the detection of the crystals was only possible by subclustering. The NMF algorithm achieved the purest Calcium oxalate spectrum: lipid bands were absent and aromatic bands smaller compared to the other two methods (Fig. 6c). The crystals are tiny and thus other components dominate the spectra in most of the pixels. So, if not known a priori and searched for with a marker band or continued with subclustering, it might be difficult to detect this layer by band integration and cluster analysis.

In contrast, the fact that aromatics play a role throughout the investigated plant surface, becomes immediately clear by integration of the strongest band at 1605 cm^−1^, (aromatic rings in conjugation with C=C/C=O; [64, 65] (Fig. 2e, f). Band integration of the neighboring bands at 1660, 1635, and 1570 cm^−1^ highlights lignin, coumaric acid and flavonoids, respectively (Fig. 2e, f). Although different distributions are derived, overall intensities must be taken with care as the overlapping bands will influence each other. Peak fitting might be a solution for such overlapping bands, but pitfalls come along with this approach [110]. If components are present at some of the pixels more “purer” or at least with changing amounts, the unmixing algorithm results in endmembers, which are characteristic for flavonoids and coumaric acid (EM 4) or lignin (EM 5) and shows their distribution (Fig. 6e, f). The high accumulation of flavonoids in the periclinal epidermal cell wall was confirmed by all three methods (Fig. 6e). The distinction of the whole epidermal layer (including the upper “aromatized” layer) was only possible by NMF (EM5 lignin and EM6 cell wall) and integration of the cellulose band at 380 cm^−1^ (Fig. 6f, g). The cellulose integration image is noisy as the band is relatively weak. Carbohydrate bands often get overlapped by aromatic bands due to the high Raman cross section of conjugated aromatic molecules [64]. Cluster analysis separates the epidermal layer into three clusters (EM3-5)—due to the changing amount of aromatics and carbohydrates (Fig. 6e–g).

Our example on the spruce needle shows that the quick band integration approach works very well as long as bands of the different components do not overlap too much, and bands are not too weak. The main component classes (waxes, lipids, aromatics, minerals, carbohydrates) can be tracked by finding marker bands. Cluster analysis groups similar spectra and results in chemically most different regions and thus does not necessarily track specific components. The NMF algorithm looks for the purest component spectra and models their distribution. The result are clear images and distinction of layers and endmember spectra coinciding best with spectra acquired from reference components (Additional file 1: Fig. S2–S8). This helped to attribute the bands to different components and their molecular vibrations (Table 1).

## Conclusions

Raman imaging of the cuticle and epidermis probed all chemical components at once in context with the microstructure and gave new insights into spruce needle surfaces:

- A crystalline wax layer, with aliphatic chains and coumaric acid aligned perpendicular to the plant surface, is distinguished from the more amorphous lipidic cuticle, which is impregnated with coumaric acid and flavonoids.
- Aromatic components are co-located with lipids (within 300 nm) in the cuticle and wax layer as even endmember spectra derived by the NMF unmixing approach showed Raman bands of both component classes.
- Calcium oxalate crystals accumulate at the interface between the lipidic cuticle and the carbohydrate rich epidermis.
- The upper periclinal epidermal cell wall is distinguished by all three data analysis approaches as a chemically different layer due to the strong Raman signals of aromatics.

The aromatic impregnation of cuticle starts in the anticlinal middle lamellae of the epidermal cells and together with the strong periclinal cell wall impregnation it is reminiscent of the casparian strip. Looking with our approaches at different developmental stages of plant surfaces will give new insights into the development of the cuticle by tracking lipid and aromatic pathways during development.

The strong impregnation of the epidermal layer offers additional protection and Raman imaging now gives a comprehensive picture of both layers as well as the Calcium oxalate interface. Future comparative studies might help to answer why some trees and their needle and leaf surfaces are more resistant to biotic and abiotic stresses than others.

## Materials and methods

### Material and preparation

Four branches of a Norway spruce tree were received from Praxmar (Tyrol, 47° 09′ N/11° 07′ E, see also [111]). The harvest took place in August 2019 and samples were immediately frozen to -20 °C after harvesting. Needles on top of the branches were selected (young needle) and a piece of the center was cut out (see also Fig. 1a). These pieces were subsequently cut into 15–20 µm thick cross sections by a cryo-microtome (CM 3050 S, Leica Biosystems Nussloch GmbH, Germany). The sections were washed with distilled water afterwards and put on a standard microscopy glass slide with a drop of distilled water, covered with a standard microscopy coverslip (0.16 mm thick) and sealed with nail polish to prevent water evaporation during Raman imaging experiments.

### Confocal Raman microscopy

We used a confocal Raman microscope (alpha300RA, WITec GmbH, Germany) with a 100 × oil immersion objective (NA 1.4, 0.17 mm with coverslip correction) (Carl Zeiss, Germany) to obtain Raman images from the aforementioned needle thin sections. A microscopical overview of every section was obtained and then suitable areas for measurement selected in the cuticle zone of the needle. A linear polarized (0°) λ_ex_ = 785 nm laser (WITec, Germany) and a λ_ex_ = 532 nm laser (WITec, Germany) were used for the experiments. The scattered Raman signal was detected with an optic multifiber (100/50 nm diameter, respectively) directed to a spectrometer UHTS30 (WITec, Germany) (600gmm^−1^ grating) and to a CCD camera (DU401DD/DU401BV, respectively) (Andor, Belfast, NorthIreland). The Control Four acquisition software (WITec, Germany) was used for control of the measurement. The laser power was set to 150 mW and integration time to 0.1 s for 785 nm experiments and to 44.7 mW and to 0.1 s for 532 nm experiments. No destructive effects of the laser on the samples were observed. A spectrum was taken every 0.3 µm to reach the maximum possible diffraction limited spatial resolution (r = 0.61 × λ/NA). The maximum theoretical spatial resolution obtainable therefore was about 342 nm for the 785 nm laser and 230 nm for the 532 nm laser. Routinely, before starting the Raman measurements, calibration of the instrument to the silicon band of 520 cm^−1^ was performed.

### Data analysis

Spectra were cropped (300–1800 cm^−1^), cosmic rays removed and the baseline corrected before calculating Raman images by integrating specific bands (univariate), cluster analysis and non negative matrix factorization (NMF) using the WITec Project plus 4.1 software (WITec, Germany). By integrating specific Raman bands a fast overview about the chemical heterogeneity was achieved. Average spectra were extracted from selected regions of the images by using an intensity threshold to include only the pixels with signal of the Raman band of interest. Cluster analysis takes into account the whole wavenumber range and segments the hyperspectral dataset in clusters according spectral similarity. As a measure for the spectral similarity Euclidean distance was chosen. The analysis was performed with 4–8 clusters, and finally the results based on 7 clusters are shown. For control, a sub-clustering into another 2 clusters was performed within every cluster (Additional file 1: Fig. S1). Cluster average spectra were extracted for detailed analysis. To find the most pure components within the dataset the unmixing algorithm non negative matrix factorization (NMF) was applied. Hyperspectral images obey a natural chemically meaningful bilinear model, the Beer–Lambert law (D = CS^T^ + E) with D as the raw Raman image, S^t^ the matrix of pure spectra, C the stretched concentration profiles and E the error [112]. Thus, an unmixing algorithm aims to retrieve the pure components or endmember spectra and their concentration profile to be displayed distribution maps. NMF was calculated based on 4 to 8 endmembers (pure components) with up to 100,000 iterations and finally the results based on 7 endmembers are shown. Average spectra based on the integration approach, based on cluster analysis as well as endmember spectra from NMF are exported into OPUS 7.5 (Bruker, Germany) for further analysis and comparison with spectra acquired from reference components.

## Supplementary Information

**Additional file 1.** Cluster analysis of spruce cuticle and reference Raman spectra of lipidic and aromatic components.

## Data Availability

The datasets used and/or analysed during the current study are available from the corresponding author on reasonable request.
